# Acute and long-term effects of adolescence stress exposure on rodent adult hippocampal neurogenesis, cognition, and behaviour

**DOI:** 10.1038/s41380-023-02229-2

**Published:** 2023-08-23

**Authors:** Alessandra Borsini, Juliette Giacobbe, Gargi Mandal, Maura Boldrini

**Affiliations:** 1 Stress, Psychiatry and Immunology Laboratory, Institute of Psychiatry, Psychology and Neuroscience, Department of Psychological Medicine, King’s College London, London, UK; 2 Department of Psychiatry, Columbia University, Molecular Imaging and Neuropathology Division, New York State Psychiatric Institute, New York, NY USA

**Keywords:** Neuroscience, Molecular biology

## Abstract

Adolescence represents a critical period for brain and behavioural health and characterised by the onset of mood, psychotic and anxiety disorders. In rodents, neurogenesis is very active during adolescence, when is particularly vulnerable to stress. Whether stress-related neurogenesis changes influence adolescence onset of psychiatric symptoms remains largely unknown. A systematic review was conducted on studies investigating changes in hippocampal neurogenesis and neuroplasticity, hippocampal-dependent cognitive functions, and behaviour, occurring after adolescence stress exposure in mice both acutely (at post-natal days 21–65) and in adulthood. A total of 37 studies were identified in the literature. Seven studies showed reduced hippocampal cell proliferation, and out of those two reported increased depressive-like behaviours, in adolescent rodents exposed to stress. Three studies reported a reduction in the number of new-born neurons, which however were not associated with changes in cognition or behaviour. Sixteen studies showed acutely reduced hippocampal neuroplasticity, including pre- and post-synaptic plasticity markers, dendritic spine length and density, and long-term potentiation after stress exposure. Cognitive impairments and depressive-like behaviours were reported by 11 of the 16 studies. Among studies who looked at adolescence stress exposure effects into adulthood, seven showed that the negative effects of stress observed during adolescence on either cell proliferation or hippocampal neuroplasticity, cognitive deficits and depressive-like behaviour, had variable impact in adulthood. Treating adolescent mice with antidepressants, glutamate receptor inhibitors, glucocorticoid antagonists, or healthy diet enriched in omega-3 fatty acids and vitamin A, prevented or reversed those detrimental changes. Future research should investigate the translational value of these preclinical findings. Developing novel tools for measuring hippocampal neurogenesis in live humans, would allow assessing neurogenic changes following stress exposure, investigating relationships with psychiatric symptom onset, and identifying effects of therapeutic interventions.

## Introduction

Adolescence is a critical developmental period characterised by intense behavioural and cognitive changes [1, 2], and crucial for establishing adult brain and behaviour health [3, 4]. Adolescence spans from post-natal day (PND) 21 to 65 in rodents (mice and rats), and from 12 to 18 years of age in humans [1]. From a behavioural perspective, adolescent rodents [2, 5, 6] and humans [2] show increased social activity [7], risk-taking [8] and impulsivity [6], compared to other age groups. Moreover, cognitive changes occurring in adolescence [9], especially social cognition and control of executive functions [10, 11], have been suggested to correspond to maturation of brain circuits that are critical for learning and memory, particularly in the hippocampus [12].

The adolescent hippocampus has more granule cells and a larger volume compared to the adult hippocampus in both rodents and humans [13, 14]. Hippocampal neurogenesis, defined as the generation of new neurons within the subgranular zone of the dentate gyrus (DG), and their integration into the granule cell layer [15], is four times higher in adolescence compared to adulthood in rodents [16] and humans [17]. Evidence generated from rodent studies suggests that neurogenesis is necessary for specific cognitive functions, including pattern separation, which is the ability to distinguish between similar but different contexts and to differentiate a threat from a neutral situation [18], as well as for antidepressant efficacy [19–22], and resilience to stress [23]. Hippocampal neurogenesis has been shown to decrease after stress in mouse models involving exposure to an intruder, intermittent feeding, social isolation, communication deprivation, and others, which can result in impaired memory, learning, and emotional regulation [24, 25].

Globally, it is estimated that 1 in 7 young people (14%) aged 10 to 19 experience mental health problems [26]. Adolescence is characterised by the presence of several psychosocial and physical stressors related to hormonal changes determining puberty, changes in body image, and evolving societal role of the individual [1]. Stress exposure can detrimentally affect neurogenesis during adolescence [25, 27, 28]. Chronic exposure to stressful situations, including psychosocial stress, social isolation, chronic unpredictable mild stress (CUMS), social instability and restrain stress, decreases adolescent hippocampal neurogenesis in mice, rats, and primates, and results in impaired hippocampal-dependent learning and memory, and depressive-like behaviours, which can last until adulthood [16, 29]. Mechanisms through which stress exposure reduces neurogenesis remain largely unknown, and may involve increased cortisol and inflammatory cytokines [30]. We have shown that exposing human hippocampal progenitor cells to cortisol or cytokines in vitro results in reduced neural progenitor cell pool, decreased neurogenesis and increased apoptosis of mature neurons [23, 31–38]. While it has been hypothesised that synaptic pruning and neurogenic changes have a role in shaping brain circuits during adolescence and consequently cognitive functions [39], few studies have addressed this question. No systematic review has examined results from the literature regarding the immediate and long-term consequence of adolescence stress exposure on neurogenesis and hippocampus-dependent cognitive and emotional functions.

It is well known that adolescence is a critical period for the onset of psychiatric disorders; with a peak/median age at onset of 14.5/20 years for obsessive compulsive disorders, 15.5/30 for stress disorders, 20.5/31 for mood disorders and 20.5/25 for schizophrenia [40], and is characterised by the presence of cognitive and emotional symptoms which persists into adulthood [41]. Changes in neurogenesis during adolescence may affect the preservation and integration of emotional memories, and the selection of memories that are maintained versus those that are filed away [42–44], which may contribute to personality development and adult mental health. As such, understanding how hippocampal neurogenesis is affected during adolescence is important, not only from a mechanistic perspective, but also for the development of novel therapeutic strategies (or for the repurposing of existing ones) targeting neurogenic mechanisms during this critical period in people suffering environmental stress exposure.

This systematic review aims to investigate acute and long-term (during adulthood) changes in hippocampal neurogenesis, neuroplasticity, and hippocampal-dependent cognitive and behavioural outcomes, in rodents exposed to stress during adolescence. In addition, this review discusses findings from studies employing pharmacological and non-pharmacological interventions as therapeutic strategies to reverse or prevent post-exposure deficits in hippocampal neurogenesis, neuroplasticity, cognition, and behaviour.

## Methods

This systematic review complies with the PRISMA (Preferred Reporting Items for Systematic Reviews) guidelines. It comprises of papers published so far until July 2023, identified across the following databases: PubMed, Embase, PsycInfo and Web of Science, which assessed hippocampal neurogenesis and neuroplasticity in adolescent rodents exposed to stress paradigms, and cognitive and depressive-like behaviour outcomes both immediately after the stress exposure as well as during adulthood. Adolescent stress models were biological, such as cortisol or cytokine injections, and behavioural paradigms, including social defeat, isolation and chronic mild stress in either rats or mice from PND21 to PND65, which corresponds to adolescence in humans [16]. Hippocampal neurogenesis was assessed by quantifying cells at different stages of maturation. In particular, cell proliferation was quantified by counting cells expressing Ki67, a marker expressed in each mitotic phase except G0, and Bromodeoxyuridine (BrdU), a marker injected either weeks and/or briefly before sacrifice. BrdU is also used to measure new-born neuron differentiation when in co-labelling with the marker NeuN, and survival. Immature neurons were quantified in those studies using the marker doublecortin (DCX) [45]. Hippocampal neuroplasticity was quantified measuring pre- and post-synaptic density proteins, long-term potentiation, as well as neurotrophic factors, which are necessary for newly generated neurons maturation and integration in existing circuits.

The complete inclusion and exclusion criteria, and the search algorithm, can be found in the Supplementary Materials, along with the PRISMA flowchart. Additionally, the studies were assessed for risk of bias, including failing to describe rodents’ baseline characteristics, random housing or blinding, following the SYRCLE guidelines for rodent studies [46] (Supplementary Table 1). The results of these studies are summarised in Table 1.

**Table 1:** Summary of studies investigating acute and long-term changes in hippocampal neurogenesis, neuroplasticity, and hippocampal-dependent cognitive and behavioural outcomes, in rodents exposed to stress during adolescence.

Study	Model/ Stressor	Sex of animals	Timing	Experimental manipulation	Timing of manipulation	Neurogenesis	Timing	Behaviour	Timing
[52]	Night-time crowding	M	~PND28	–	–	= BrdU (Cell survival and proliferation) in SGZ inj. PND21 and PND42 = BDNF in HIPP	~PND42	–	–
[56]	Restraint stress	M	PND42–56	–	–	= BrdU/DCX (Cell proliferation) inj. PND54–56	PND56	= immobility (FST) = anhedonia (SPT)	PND56
[50]	Social defeat stress	M	PND24–34	Mifepristone (20 and 40 mg/kg ip)	PND24–34	↓ Ki67 in SGZ ↓ BrdU (Cell proliferation), inj PND34 in SGZ	PND35	= immobility (FST) = anhedonia (SPT)	PND35
						↓ BrdU/NeuN (Cell survival), inj. PND35–38 in SGZ, reversed by mifepristone	PND63		
[49]	Social instability	F	PND30–45	–	–	= Ki67 ↓ BrdU (Cell survival and proliferation) inj. PND43–45 in SGZ and GCL	PND49	= Memory (SLR)	PND47–48
[51]	CORT administration	F	PND28–48	–	–	↓ BrdU (Cell proliferation) inj. PND46–48 in GCL = BrdU/NeuN (Cell survival), inj. PND28–30 in GCL	PND48	↑ immobility (FST) = anhedonia (SPT)	PND48
[47]	Social defeat stress	M	PND30	–	–	↓ BrdU (Cell proliferation) in dHIPP inj. PND42 ↓ DCX in DG	PND42	–	–
[48]	Social instability stress +/− social defeat or communication deprivation stress	M	PND28–46	–	–	↓ BrdU (Cell proliferation) inj. PND47 in social defeat stress only	PND47	↓ latency to immobility (FST) in communication deprivation stress but not social defeat stress	PND43–46
				Rest/comfortable conditions	PND43–66	= BrdU (Cell proliferation), inj. PND66	PND67	= immobility (FST)	PND63–66
[53]	Social isolation	M	PND21–49	–	–	= BrdU (Cell proliferation), inj. PND48	PND49	–	–
						↓ BrdU/NeuN (Cell survival), inj. PND21 in SGZ, GCL	PND49		
			PND21–49	Fluoxetine	PND35–49	↓ BrdU/NeuN (Cell survival), inj. PND21, **X** by Fluox, in GCL	PND49		
			PND21–49	Fluoxetine	PND35–56			↓ time spent in target quadrant (MWM), **X** by Fluox	PND49–56
[54]	Social instability	M	PND30–45	–	–	↑ Ki67	PND33	–	–
						= Ki67 ↑ DCX	PND46	= Memory (SLR) = Recognition (NOR)	PND46–49
						= Ki67 ↑ DCX = SYN ↑ CamKIIα	PND74–75	↓ Memory (SLR) = Recognition (NOR)	PND70–73
[57]	i.c. IL-1beta injection	M	PND28	–	–	↓ DCX in GCL ↓ branch points on DCX+ cells = neurite length	PND63	= pattern separation	PND49–58
								= object recognition	PND59
								= spontaneous alternation	PND60
[55]	CMS	M	PND28–42	–	–	= DCX in dHIPP, vHIPP	PND42	–	–
						↑ DCX in vHIPP	PND65		
[63]	CORT	M	PND29–49	–	–	= PSD95 ↑ mature BDNF	PND51	= anhedonia (SPT) = recognition (MWM)	PND50 PND50–55
						= PSD95 = mature BDNF	PND78	= anhedonia (SPT) = recognition (MWM)	PND77
								↑ learning (MWM)	PND77–87
[62]	CORT	M	PND29–49	–	–	↓ PSD95 ↑ mature BDNF	PND51	↑ anhedonia (SPT) ↑ recognition (MWM)	PND49,55 PND50–56
[60]	CUMS	F	PND28–61	–	–	↓ PSD-95, SYN in CA1 and DG ↓ neurons on CA1 and DG	PND62	↑ anhedonia (SPT)	PND57
[64]	Isolation	M	PND30–35	vehicle, adinazolam (10 mg/kg), MK-801 (0.3 mg/kg), or tianeptine (10 mg/kg)	PND40–55	= SYN HIPP with stress ↑ SYN by adinazolam, MK-801, or tianeptine alone	PND60	–	–
[76]	Isolation	M and F	PND30–35	–	–	↑ spinophilin in male CA3 ↓ spinophilin in female CA3	PND36	↓ latency to immobility in females on day 1 (FST) ↓ latency to immobility in males on day 2 (FST)	PND36–37
[69]	Restrain stress	M and F	PND21	–	–	↑ Arc (females only) ↓ Erg1 (males only)	PND21	–	–
[65]	Juvenile stress	M	PND27–29	–	–	↑ a-NCAM-L1 in DG and CA1	PND33	–	–
						↑ a-NCAM-L1 in DG	PND63		
[66]	Juvenile stress	M	PND27–29	–	–	↑ a-PSA-NCAM in DG and CA1	PND33	–	–
						↑ a-PSA-NCAM in DG and CA1	PND63		
[67]	Peripubertal stress (elevated platform, predator odour)	M	PND28–42	–	–	= PSA-NCAM in DG	PND55	= exploring new object (NOT)	PND48
								= MWM	PND55
						↑ PSA-NCAM in DG	PND90	↑ exploring new object (trend, NOT) ↑ time to find target platform (MWM)	PND83-PND90
[61]	Social defeat stress	M	PND35–44	–	–	= CA1 spine density ↑ long-thin spines ↓ PSD95 in spines ↓ stubby spines	PND45	↑ immobility (TST)	PND45
[79]	Chronic physical stress	M	PND28–55	–	–	↑ Volume of CA1 = Volume in CA3, DG; neuron number; neuronal soma size	PND56	↓ escape latency (MWM) = time spent in target quadrant (MWM)	PND56
						↓ Volume of CA1, CA3, DG ↑ Neuronal soma size in CA1, DG = Neuron number	PND76	↑ escape latency (MWM) ↓ time spent in target quadrant (MWM)	PND76
	Chronic social stress		PND28–55	–	–	↑ Volume of CA1 = Volume of CA3, DG; neuron number; neuronal soma size	PND56	↓ escape latency (MWM) = time spent in target quadrant (MWM)	PND56
						= Volume of CA1, CA3, DG = Neuron number ↑ Neuronal soma size in CA1, DG	PND76	= escape latency (MWM) = time spent in target quadrant (MWM)	PND76
[77]	Chronic restraint stress	M and F	PND20–41	–	–	↓ apical dendritic length ↓ branch points in CA3	PND42	↑ anhedonia (SPT)	PND40–41
								↓ immobility (FST)	PND42
[80]	Chronic variable physical stress (in animals more or less susceptible to stress)	M	PND28–41	–	–	↑ BDNF mRNA in CA3 and DG in animals less sensitive to stress ↑ mossy fibre terminal field volumes in animals less sensitive to stress ↓ mossy fibre terminal field volumes in animals more sensitive to stress	PND42	↑ immobility (FST)	PND42
[81]	Acute stress	M and F	PND38	–	–	↑ neurons in DG and CA1 ↑ BDNF in HIPP	PND42	↑spatial learning (MWM)	PND38–42
[78]	Restraint stress	M	PND21–35	–	–	= number of dendritic spines ↑ dendritic spine density in dHIPP (PND38) ↓ dendritic spine density in HIPP (PND50, 68)	PND38,50,68	–	–
[70]	Acute stress (elevated platform)	M	PND14–28	Some exps on behaviour + intracranial capsaicin	Intracranial capsaicin ≈ 6w	↓ LTP, reversed by capsaicin on slices ↑ LTD, reversed by capsaicin on slices	PND14–28	↓ recognition (MWM) ↓ time spent in target quadrant (MWM), **X** by Capsaicin	PND14–28
[74]	Isolation	M	PND22–50	Slices exposed to K-252a (serine inhibitor, also blocks Trk tyrosine kinase)	K-252a	↑ LTP, normalised by K-252a on slices ↑ BDNF	PND50	↑ latency to approach and begin eating food (novelty-suppressed feeding test) = food intake (novelty-suppressed feeding test)	~PND51
[72]	Acute stress (restraint elevated platform, forced swim)	M	PND28–30	–	–	↓ LTP in dHIPP ↑ LTP in vHIPP	PND31	–	–
						= LTP in dHIPP and vHIPP	PND38		
						= LTP in dHIPP and vHIPP	PND52		
[73]	Restraint stress	M	PND21–28	Ro25–6981 (GluN2B or NR2B subunit inhibitor)	PND21–28 (30 mins prior to restrain stress)	↓ LTP ↑ LTD, reversed by GluN2B inhibitor ↓dendritic density in CA3	~PND40–45	↓ learning (MWM)	PND29-35
								↓ recognition (NORT), reversed by GluN2B inhibitor	~PND36–37
[71]	Acute Stress	M	~PND30, 45	–	–	↓ LTP maintenance	~PND30, 45	–	–
[75]	Acute unpredictable and inescapable restraint-tailshock stress	M and F	PND33–37	–	–	↑ LTD (males only)	PND37	–	–
[85]	Social instability stress	M	PND30–45	ω-3 PUFAs and vit A enriched diet vs control diet	PND30–45	↓ BDNF, **X** by diet	PND50	↓ discrimination (NORT), **X** by diet ↑ anhedonia (SPT), **X** by diet	PND45–50
						↓ BDNF, **X** by diet	PND70	↓ discrimination (NORT), **X** by diet ↑ anhedonia (SPT)	PND70–75
[82]	Restraint stress	M and F	PND31–38	OXY (2 µg/kg, intranasally)	PND31–38	= BDNF with stress alone ↑ BDNF with stress + OXY	PND45	↑ time in opposite quadrant ↓ time in target quadrant (MWM) Both partially reversed by OXY	PND39–44
[68]	Social defeat and psychological stress	M	PND45–46	–	–	↑ BDNF, Arc, Carp, Tieg mRNA in HIPP	PND46	–	–
[83]	CMS	M	PND45–60	–	–	= BDNF mRNA	PND60	↓ latency to immobility (FST)	PND60
[84]	Isolation and footshocks	M and F	PND30–60	–	–	↓ BDNF in HIPP in males	PND60	↑ anhedonia (SPT) in males	PND60–62

*PND* postnatal day, *BrdU* bromodeoxyuridine, *BDNF* brain-derived neurotrophic factor, *DCX* doublecortin, *NeuN* neuronal nuclear antigen, *Arc* activity Regulated Cytoskeleton Associated Protein, *Erg1* early growth response protein 1, *Carp* calcium/calmodulin dependent protein kinase (CaMK)-related peptide, *Tieg1* transforming growth factor-βinducible early gene, *CaMKIIα* calcium/calmodulin-dependent kinase II aplha, *PSD-95* post-synaptic density protein 95, *NCAM-LI* neural cell adhesion molecule *L1, PSA-NCAM* polysialylated neuronal cell adhesion molecule, *ω-3 PUFAs* ω-3 polyunsaturated fatty acids, *Vit A* vitamin A, *OXY* oxytocin, *SYN* synaptophysin, *LTP* long-term potentiation, *LTD* long-term depression, *CORT* corticosterone, *CUMS* chronic unpredictable mild stress, *CMS* chronic mild stress, *SPT* sucrose preference test, *FST* forced swim test, *MWM* Morris water maze, *NOR* novel object recognition test, *SLR* spatial location recognition, *NORT* novel object recognition task, *NOT* novel object test, *OFT* open field test, *SGZ* subgranular zone, *MOL* molecular layer, *GCL* granule cell layer, *HIPP* hippocampus, *dHIPP* dorsal hippocampus, *vHIPP* ventral hippocampus, *DG* dentate gyrus, *CA1* cornu ammonis 1, *CA3* cornu ammonis 3, *ip* intraperitoneally, *inj* injection, *M* male, *F* female, *NM* not mentioned, ↑ increase, ↓ decrease, = no change, *X* abolished.

## Results

In total, 905 studies were extracted and 37 of these met our inclusion criteria (Supplementary Fig. 1). Inclusion criteria for the studies were: using rodents who underwent stress exposure biological or behavioural paradigms during adolescence, and assessed hippocampal neurogenesis and neuroplasticity, together with behavioural and cognitive outcomes immediately after stress exposure and later during adulthood. Specifics about timings and type of adolescent stress paradigms, hippocampal neurogenesis measures, neuroplasticity markers, and cognitive and behavioural assessments, are reported in Table 1.

### Acute cellular and behavioural outcomes of adolescence stress exposure

#### Hippocampal neurogenesis and hippocampal-dependent cognitive and behavioural functions

Seven studies assessed cell proliferation quantifying cells BrdU positive (+) cells in single-labelling, which detected any type of proliferating cells [47–53]. Among these studies, five reported decreased cell proliferation in stress exposed rodents, independently of the type and length of stressor, which was social defeat (PND24–34 [50] and PND30 [47]), social instability (PND30–45 [49], PND28–46 [48]), or cortisol administration (PND28–48) [51]. In contrast, two studies observed no changes in cell proliferation upon exposure to crowding at PND28 [52] or social isolation at PND21–49 [53].

Two studies found an association between fewer BrdU+ proliferating cells and increased depressive-like behaviour at the forced swim test [48, 51], but another study did not [50]. Another study showed no link between lower proliferation and spatial memory, measured using object recognition and spatial location tests [49].

Three studies [40, 41, 53] quantified Ki67+ cells, which are in any phase of mitosis except G0 and showed that early on Ki67+ cells are found increased at PND33 [54] and decreased at PND35 [50], and there was no longer any change at PND46 [54] or PND49 [49]. Types of stress were social defeat or social instability, and the timing of stress exposure was earlier (PND24–34) in the study that found decreased Ki67+ cells [50], and later (PND30–45) in the study reporting a Ki67+ cell increase [49, 54]. No relationship with memory (spatial and object recognition) or depressive-like behaviour (forced swim and sucrose preference test) was found in the study reporting decreased proliferation after social defeat at PND24–34 [50] and social instability stress at PND30–45 [49, 54].

Five studies measured the effects of adolescent stress exposure on number of cells expressing doublecortin (DCX+) [47, 54–57], which has been largely used to assess numbers of neuroblasts or immature neurons in rodents, human, and non-human primates [58], although recent studies have questioned its specificity as neurogenesis marker [16, 59].

After exposure to social defeat fewer DCX+ cells were reported in rats at PND42, and the adolescent defeated rats more frequently initiated play behaviour but adopting submissive postures, while once they became adults, they coped behaviourally and physiologically better with a similar exposure to an aggressive male rat than unstressed controls [47].

Fewer DCX+ cells and reduced neurite branching on hippocampal neurons were also observed at PND63 after treatment with interleukin (IL)-1beta (IL1β), without an effect on performance in pattern separation, novel object recognition or spontaneous alternation in the Y maze [57].

An increase in DCX+ cells was reported at PND46 after social instability [54], and at PND65 after chronic mild stress (CMS) [55], while there was no change in DCX+ cells after CMS at PND42 [55] or restraint stress at PND56 [56]. No change in pattern separation, memory (object recognition, spatial recognition), or depressive-like behaviour (forced swim and sucrose preference test) was found when increased or unchanged DCX+ cell number were reported after stress [54, 56, 57].

New-born neurons quantified by counting cells co-localising for BrdU and the neuronal marker NeuN (BrdU/NeuN+), after BrdU injections three and four weeks before sacrifice [50, 51, 53] were fewer after chronic social defeat exposure during PND24–34 [50] and social isolation at PND21–49 [53]. These effects were reversed by mifepristone, a glucocorticoid receptor (GR) antagonist [50], and the antidepressant fluoxetine [53]. A study did not show any difference in new-born neuron number in rodents exposed to chronic cortisol treatment at PND28–48 [51].

The study showing fewer BrdU/NeuN+ new-born neurons after social isolation at PND21–49, found altered spatial memory and emotion-related behaviours in juvenile mice [53]. While two studies showing fewer BrdU/NeuN+ new-born neurons after social defeat at PND24–34 [50] or no difference in BrdU/NeuN+ neurons after cortisol treatment at PND28–48 [51], did not find an effect of these exposures on depressive-like behaviour measured with the sucrose preference test [50, 51].

#### Neuroplasticity and hippocampal-dependent cognitive and behavioural outcomes

Ten studies measured changes in synaptic density and neuroplasticity after adolescent stress exposure [60–69]. Post-synaptic density 95 (PSD95) [60–62] and the pre-synaptic synaptophysin (SYN) [60] were decreased upon exposure to chronic stress with either cortisol, CMS or social defeat stress during PND29–49 [62], PND28–61 [60] and PND35–44 [61]. In contrast, two studies that used cortisol or social isolation chronic stress exposure during PND29–59 and PND30–35, reported unaffected PSD95 and SYN levels [63, 64].

Decreases in PSD95 were accompanied by depressive-like behaviour, measured using the sucrose preference test [60, 62]. The two studies that used cortisol or social isolation reporting unaffected PSD95 and SYN levels also found no change in the sucrose preference test and Morris water maze [63, 64].

Additionally, proteins expressed in the presence of neuroplastic activity, such as polysialylated-neural cell adhesion molecule (PSA-NCAM) and neural cell adhesion molecule L1 (NCAM-L1), were increased in a study upon exposure to auditory fear conditioning soon after the stressful experience and during adulthood, suggesting alteration of the normal maturational decrease in L1 expression and therefore delayed maturation of the limbic system [65]. Another study exposed juvenile rats to variable stress, delivering a different stressor every day for 3 days, forced swim, elevated platform, and foot shock or restraint stressors (at PND27–29) [66] and found missing development-related decrease in PSA-NCAM to NCAM expression ratio in the basolateral amygdala, in the CA1 and dentate gyrus regions of the hippocampus, and in the entorhinal cortex, with an increase in the polysialylation of NCAM soon after exposure and in adulthood. A third study of exposure to chronic peripubertal stress protocol consisting of two different fear-inducing stressors: exposure to a synthetic fox odour, and elevated platform at PND28–42 found that peripubertal stress led to changes in emotional and glucocorticoid reactivity to novelty exposure, as well as in the expression levels of the plasticity molecule PSA-NCAM in the hippocampus. [67]. Similarly, other neuroplastic proteins, such as the immediate early gene Arc, involved in the consolidation of memories, were increased in two studies upon exposure to restraint and social defeat stress at PND21 and PND45–46, respectively [68, 69], whereas Erg1, involved in learning and memory, was decreased after social defeat only in male rodents [69].

Six studies showed dysfunctions in long-term potentiation (LTP) and long-term depression (LTD) in the hippocampus, which are plasticity processes associated with the strengthening or weakening of synaptic connections, respectively [70–75]. Four studies reported decreased LTP after acute restraint stress during PND14–28 [70], PND28–30 [72], PND21–28 [73] and PND30 [71], whereas one study reported increases in LTP after chronic social isolation at PND22–50 [74]. Three of these studies observed increases in LTD after acute restraint stress (PND14–28, PND21–28, PND33–37) only in male rodents [70, 73, 75]. Of note, the changes in LTP and LTD, during the stress challenge, were reversed by treatment with antidepressant-like compounds capsaicin [70], an agonist of the transient receptor potential vanilloid subtype (TRPV1), and Ro25–6981 [73], an inhibitor of the glutamate N-methyl-D-aspartate (NMDA) receptor GluN2B subunit. Moreover, impairment in learning, spatial memory and recognition, measured using the Morris water maze test and the novel object recognition test, were observed [70, 73], and were reversed by both capsaicin and the GluN2B subunit inhibitor [70, 73].

Two studies found a decrease in LTP after acute restraint stress during PND28–30 [72], and PND30 [71], but no cognitive or behavioural changes were measured.

In contrast, two studies found an increase in LTP after chronic social isolation at PND22–50 [74] or after acute restraint stress at PND33–37 in male rodents [75], with one study founding behavioural changes, particularly an increase in latency to approach and begin eating food, measured through the novelty-suppressed feeding test, but no changes in overall food intake [74]. The other study did not measure neither cognition nor behaviour [75].

Six studies reported that dendritic formation, density and morphology were disrupted after adolescent stress [61, 73, 76–79]. A marker of dendrite formation, spinophilin, was increased in males after social isolation, but decreased in females upon exposure to social isolation at PND30–35, and was associated with a decrease in latency to immobility during forced swim test, considered a measure of behavioural despair or learned helplessness, in both males and females [76]. Four studies reported that exposure to social defeat (PND35–44) and chronic restraint stress (PND20–41, PND21–35, PND21–28) reduced dendritic spine density and detrimentally affected their morphology (length and size) [61, 73, 77, 78]. Dendritic spine density and morphology changes were accompanied by memory deficits, measured using the Morris water maze test, and depressive-like behaviours, measured with the forced swim test and the sucrose preference test [73, 77]. Chronic physical stress decreased mossy fibres, axons of DG granule cells that project within the hilus and stratum lucidum, and innervate hilar cells and CA3 pyramidal cells and increased hippocampus *Cornu Ammonis* (CA)1 volume in both wild type (exposed at PND28–55) and in variable physical stress sensitive rats (exposed at PND28–41) [79, 80]. The rats were classified on the basis of their locomotor reactivity to novel objects, which has been associated with sensitivity to stress [80]. These changes were accompanied by spatial memory deficits, measured using the Morris water maze test, and increased depressive-like behaviour, measured with the forced swim test [79, 80]. Overall, deficits in dendrite formation and changes in their density and morphology were associated with memory dysfunctions and depressive-like behaviour.

Ten studies investigated changes in brain-derived neurotrophic factor (BDNF), which is involved in promoting cell proliferation growth, and survival [62, 63, 68, 74, 80–85]. Six of the 10 studies reported increases in BDNF protein and mRNA expression upon exposure to chronic cortisol treatment (PND29–49), physical stress (PND28–41), acute restraint stress (PND38), and social defeat stress (PND45–46) [62, 63, 68, 74, 80, 81]. Two studies showed BDNF protein decreases upon social instability stress (PND30–45) and social isolation (PND30–60) only in male rodents [84, 85]. Two other studies showed no differences in BDNF protein and mRNA expression after exposure to crowding (PND28), restraint stress (PND31–38) and CMS (PND45–60) [82, 83]. In terms of cognition and behaviour, a study found that BDNF reduction was associated with a disruption in cognitive performance, and increased depressive-like behaviours, measured with the sucrose preference test, which was reversed by supplementing rodents with an omega-3 fatty acids and vitamin A enriched diet during the stress challenge (PND30–45) [85]. Three studies reported a post-stress decrease in memory, measured with the Morris water maze test, and an increase in depressive-like behaviour, measured with the forced swim test and the sucrose preference test, even when they found either no change or increased BDNF levels [82–84]. However, two other studies found that increased BDNF after stress was associated with better spatial learning in the Morris water maze [63, 81]. Taken together, these findings show that dietary interventions reverse stress-induced detrimental changes in hippocampal neuroplasticity, cognitive function and behaviour.

### Delayed cellular and behavioural outcomes of adolescence stress exposure

#### Hippocampal neurogenesis, cognitive functions, and behavioural outcomes

Only two of the 37 studies assessed neurogenesis outcomes in adulthood after adolescence stress exposure [48, 54]. The first study showed an initial increase in cell proliferation (Ki67+ cell number) at PND33, upon exposure to social instability stress (PND30–45), which did not last over time and disappeared at PND74–75, and reported that these rodents had spatial memory impairments in adulthood [54]. The second study showed that, impaired proliferation (BrdU+ cell number) and depressive-like behaviour (forced swim test) observed at PND47 after social instability stress exposure (during PND28–46), were no longer present in adulthood (PND67) [48]. Together, these studies show that cell proliferation decreases close to the stress exposure during adolescence, do not persist, as neurogenesis is restored and depressive-like behaviour disappear after a period of non-exposure, at least in resilient rodents.

Studies reporting fewer DCX+ cells at PND42 after social defeat [47] showed more submissive behaviour in adolescence, although rats were able to cope once they got to adulthood.

#### Neuroplasticity and hippocampal-dependent cognitive and behavioural outcomes

Out of the 37 studies, six of them assessed neuroplasticity outcomes in adulthood [54, 63, 67, 78, 79, 85]. Two studies found that dendritic spine density [78] and CA1 volume [79] decreased over time after adolescent restraint stress (PND21–35) and chronic physical stress (PND28–55), when comparing PND56 with PND76 [79], and PND38 with PND68 timepoints [78]. Reduced BDNF protein levels were found to either normalise or remain decreased into adulthood at PND78 [63] and PND70 [85], after adolescent exposure to cortisol (PND29–49) [63] and social instability stress (PND30–45) [85]. One study observed increased expression of the plasticity marker PSA-NCAM at PND90, which was not present during adolescence (PND28–42) [67]. Another study reported that levels of PSD95, which were unchanged after adolescent exposure to cortisol at PND51, remained the same at PND78 [63]. With regards to changes in cognitive function, measured with spatial location recognition and spatial memory, and behavioural outcomes, measured with the sucrose preference test, these either persisted or developed during adulthood [54, 63, 67, 79, 85].

Another study exposed juvenile rats to variable stress, delivering a different stressor every day for 3 days, forced swim, elevated platform, and foot shock or restraint stressors (at PND27–29) [66] and found reduced novel-setting exploration and impaired two-way shuttle avoidance learning in adulthood.

Interestingly, only cognitive but not behavioural changes occurring during adulthood were prevented by the dietary supplements (omega-3 fatty acids and vitamin A), that ameliorated cognitive function and in depressive-like behaviours during adolescence, even if tehre were continuously administered since adolescence (PND30–75) [85]. However, only cognitive but not behavioural changes occurring during adulthood were prevented by the dietary supplements, administered since adolescence (PND30–75). Together, these studies found that detrimental effects on neuroplasticity, cognitive functions and behaviour can either persist or develop in adulthood as a consequence of stress exposure during adolescence, and demonstrate the beneficial role of nutritional interventions in preventing these effects.

## Discussion

We provided the first *systematic* review of the available literature investigating acute and long-term changes in hippocampal neurogenesis, neuroplasticity and hippocampal-dependent cognitive and behavioural outcomes occurring in rodents exposed to stress during adolescence. Overall, studies found a reduction in hippocampal cell proliferation (BrdU+ cells only) associated with increased depressive-like behaviours in rodents exposed to stress challenges, however a reduction in the number of new-born neurons was not accompanied by changes in cognition and behaviour. In addition, studies observed alterations in neuroplasticity, including a decrease in pre- and post-synaptic markers, dendritic spine length and density, and in synaptic potential. Changes in neuroplasticity were accompanied by cognitive impairments, such as a decrease in learning and memory, and by an increase in depressive-like behaviours. The detrimental effects of stress on cell proliferation, cognition and depressive-like behaviour that were observed during adolescence had variable impact in adulthood. Interestingly, treatment with antidepressants, glutamate receptor inhibitors or GR antagonists (during adolescence), or omega-3 fatty acids and vitamin A supplements administered (during both adolescence and adulthood), prevented or reversed those detrimental changes.

**Fig. 1:**
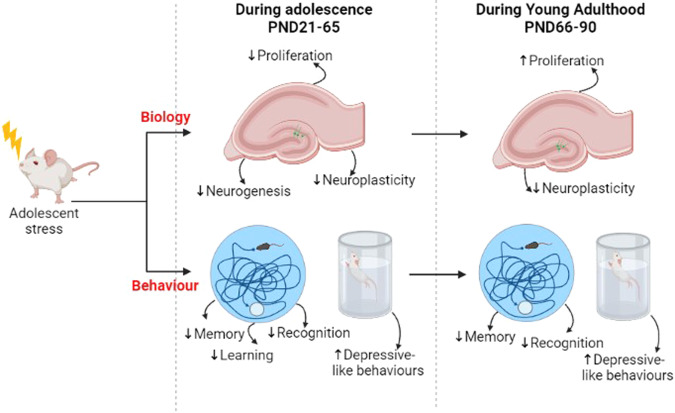
Schematic representation of the effects of stress exposure on rodent hippocampal neurogenesis, neuroplasticity, cognitive functions and depressive-like behaviours during adolescence (PND 21–65) and adulthood (PND66–90). Changes (increase or decrease) in the aforementioned outcomes are indicated with arrows. Legend: increase (↑) or decrease (↓).

In adolescent rodents exposed to stress challenges, results show a significant reduction in hippocampal cell proliferation (BrdU+ cells) and a concomitant increase in depressive-like behaviours (Fig. 1), measured with the forced swim test and the sucrose preference test. In particular, rodents exposed to social instability, social defeat stress or cortisol administration between PND24 and PND49 had a lower number of proliferating cells within the hippocampus [47–51], which were cell that were not characterised in terms of their phenotype. Blunted cell proliferation was independent of stress type (social defeat, social instability, or cortisol administration), duration (acute or chronic), and time of brain tissue collection (immediately after the stress challenge to up to 12 days after). Moreover, among these the one that did not find any depressive-like behaviour used social instability [48] as stress paradigm, while those that found an increase in depressive-like behaviour, used cortisol treatment [51] and social defeat stress [50]. Result indicate that the time of behavioural testing did not matter, either immediately after the challenge [48, 51] or a day after the end of the stress challenge [50], as in both cases studies found depressive-like behaviour, except for the social instability exposure. Additionally, the longer stress exposure appeared to induce increased immobility in the forced swim test, considered indicative of behavioural despair or learned helplessness, and a proxy for depressive behaviour, in animals exposed between PND28–46 [48] and PND28–48 [51], whereas, shorter exposure during PND24–34 induced no change in immobility [50]. This suggests that any stress can decrease cell proliferation, and that the type and length of stress affects how these result in depressive-like behaviour.

Other studies using social instability [40, 41], social defeat [47, 50] and using Ki67 as a marker of cells in any phase of mitosis except G0, found either a decrease, increase or no change in the expression of this marker, and no changes in recognition [49, 50, 54]. These findings are inconsistent with previous evidence generated for BrdU and could be explained by the fact that while BrdU is detected throughout the cell lifetime, Ki67 is expressed only during mitosis [86]. With respect to differences observed among the studies using Ki67, one found increased Ki67 expression immediately after stress at PND33 [54], another found decreased Ki67 at PND35 [50], and the third found no change later on at PND49 [49], suggesting that proliferation may surge immediately, decrease right after and then become stable again after two weeks. Studies that found no change or increased Ki67 expression and no change in recognition or depressive-like behaviour used social instability stress at PND30–45 [49, 54], while the one that found decreased proliferation used social defeat stress and at an earlier time PND24–34 [50], which suggests that age at stress exposure, and type of stress contribute to proliferative reactions to stress. Again, these proliferating cells could have been any type of cell, including not only neural progenitors, but also vasculature, microglia, or other types of glia.

Two studies observed a reduction in new born neurons, identified as cells co-labelling for the neuronal marker NeuN and BrdU (BrdU/NeuN) (Fig. 1) [50, 53], however one study observed no change in newly born neuron survival using the same markers [51]. Differences between the studies were the nature of stress challenge, intervals of BrdU injections, and cells counting method. While studies finding reduced cell survival had utilised social stress paradigms (social defeat stress [50] and social isolation [53]) and cells were counted four weeks after BrdU injection, the study which observed no change had administered rodents with cortisol and the cells were counted 3 weeks after BrdU injections, possibly too soon for neuronal differentiation. Furthermore, there were inconclusive findings regarding changes in the number of neuroblasts or immature neurons, detected by quantifying the expression of doublecortin (DCX) [47, 54–57]. While two studies showed a decrease in DCX positive cells after stress [47, 57], two other studies found the opposite [54, 55]. Again, the studies used different stress types and durations: chronic biological (IL1β injection, at PND28) [57] and acute social (social defeat stress, at PND30) [47], versus chronic social stress challenges (social instability, PDN30–45 [54]; CMS, PND28–42 [55]). In the acute social and chronic immune challenge, the number of DCX immature neurons decreased [47, 57]. In line with these findings, our in vitro experiments showed that exposing human hippocampal progenitor cells to acute immune challenge with IL1β, reduced the number of immature neurons [36]. The number of immature neurons generated by progenitor cells appears to be affected by the type and duration of the IL1β insult.

An opposite trend was observed when using chronic social stress [54, 55], where the animals might have had time to adapt to the social stress and develop coping strategies. We reported that resilient individuals, with early life adversity exposure before age 16 and no psychopathology lifetime, have more granule cells and a larger DG than suicide decedents with and without early life adversity exposure, and non-exposed controls [87]. This is in line with the possibility that resilient mice within the same strain might have more neurogenesis and more granule neurons, supporting their effective coping strategies.

Novel object recognition, sucrose preference, and forced swim test did not show any deficit associated with changes in DCX+ and BrdU/NeuN+ cell numbers, irrespective of the stress challenge used (restrain stress, social instability, cortisol or IL1β injection) [50, 51, 54, 56, 57].

Studies investigating markers of neuroplasticity found decrease expression of synaptophysin [60] and PSD95 [60–62], markers of pre- and post-synaptic plasticity, and reduced dendritic spine density [61, 73, 77, 78], and synaptic potential [70–73, 75] (Fig. 1). The reduction in dendritic spine density was independent upon the type of stress challenge, as studies using restrain stress [73, 77, 78] and those using social defeat stress [61]).

Importantly, reduction in LTP and increase in LTD measured using electrophysiology were accompanied by decreased object recognition and spatial memory as measured by Morris water maze and the novel object recognition test [70, 73], observed immediately after the last day of stress, and independently of whether rodents were exposed to either an acute [70] or chronic stress challenge [73].

Decreased PSD95 and synaptophysin were accompanied by depressive-like behaviours, measured using the sucrose preference test [60, 62] (Fig. 1), independently of the type of stress challenge used, either biological (cortisol) [62] or psychological (CUMS) [60] that were applied chronically (PND29–49, PND28–61) [60, 62]. Therefore, future studies should test the differential effects of acute and chronic stress exposure on different types of neuroplasticity as well cognitive and behavioural functions, during adolescence and later in life.

Some of the biological, cognitive and behavioural effects observed during adolescence either worsened or persisted during adulthood, especially number of proliferating cells identified using Ki67 [54], dendritic spine density [78], hippocampal volume [79], and levels of neurotrophic factor BDNF [84, 85] (Fig. 1). Others observing no behavioural or physiological effects persisted into adulthood [47], showing that the final consequence of childhood adversity depends on how well early and later life environmental challenges match each other (“match-mismatch hypothesis”). In fact, socially stressed adolescents were resilient to early stress exposure if they were socially housed afterwards, which granted them the ability to recover [47]. Studies that observed increased Ki67 (at PND33) and hippocampal volume (at PND56) during adolescence upon exposure to social instability, chronic physical stressors (such as forced swim or cold exposure) or social stressors (including, loud noise, novel environment or crowding), found a reduction in Ki67 and hippocampal volume during adulthood (Ki67 at PND74–75; hippocampal volume at PND76) [54, 79]. Similarly, another study, which observed a decrease in BDNF levels during adolescence (at PND50) found these levels to remain decreased during adulthood (at PND70) [85]. Together with BDNF, negative changes in learning, object discrimination and performance in the Morris water maze test, which were previously observed during adolescence, remained negatively affected also during adulthood [85]. Overall, these findings are quite striking as so far only a limited number of studies have examined changes in neurogenesis, recognition, memory and behaviour during adolescence in models of depression, as well as their persistence later in life. Of note, these results correspond to findings in humans, which show that cancer treatment in children and adolescents with brain radiation, which ablates hippocampus neurogenesis [88, 89], produces long-term cognitive impairments along with depressive symptoms [90]. This is fundamentally important as it proposes adolescence as a perfect time for therapeutic interventions.

**Fig. 2:**
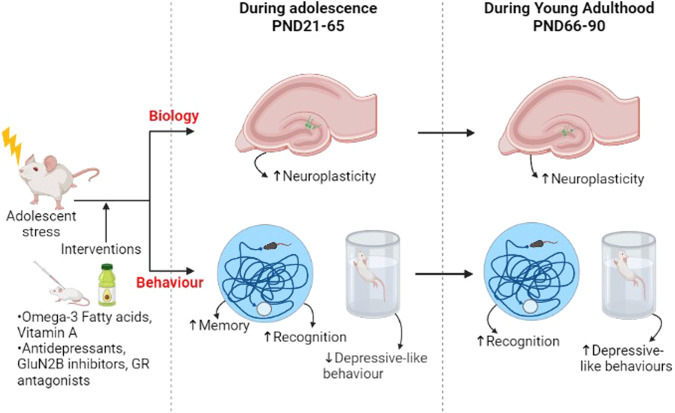
Schematic representation of the beneficial effect of treatment with either omega-3 fatty acids, vitamin A, antidepressants, glutamate receptor inhibitors or glucocorticoid receptor antagonists on rodent hippocampal neurogenesis, neuroplasticity, cognitive functions and depressive-like behaviours during adolescence (PND21–65) and adulthood (PND66–90). Changes (increase or decrease) in the aforementioned outcomes are indicated with arrows. Legend: increase (↑) or decrease (↓).

Notably, treatment during adolescence with either antidepressants, glutamate receptor inhibitors or GR antagonists reversed the detrimental effect of stress previously observed on neuronal survival (NeuN), neuroplasticity (decrease in LTP), and recognition [50, 53, 73] (Fig. 2). This was independent of the type of stress, duration of stress, or type and duration of pharmacological treatment [50, 53, 73]. In line with these findings, extensive evidence has demonstrated that functional hippocampal neurogenesis is necessary for antidepressants to exert their beneficial properties on both cognition and behaviour [41, 91, 92]. In particular, the time course of maturation of newly generated neurons in the DG, which is generally consistent with the delayed onset of therapeutic action of antidepressants, and the unique physiological properties (plasticity and excitability) of adult-born dentate granule neurons qualify adult hippocampal neurogenesis as a fundamental antidepressant target [41, 91, 92]. At present, one neurogenic and neurotrophic compound called NSI-189 phosphate (NSI-189), whose antidepressant activity is monoamine-independent, has been tested in adult patients with depression (phase 2b trial). Results showed significant improvements in cognitive function and a reduction in depressive symptoms after 12 weeks of oral treatment [93]. These findings are quite interesting and suggest that pharmacological compounds targeting neurogenesis could be valid alternative therapeutic approaches for patients with depression experiencing neurogenic and cognitive alterations.

In addition to pharmacological treatments, nutritional intervention with omega-3 fatty acids and vitamin A since adolescence (PND30–45), reversed the decrease in BDNF level and object discrimination performance, previously observed during adolescence (at PND50), but also prevented their persistence during adulthood (at PND70) (Fig. 2) [85]. The omega-3 fatty acids and Vitamin A dietary supplements could prevent the decrease in sucrose preference shortly after the intervention in adolescence (PND45), however this preventative effect did not persist during adulthood (PND70) [85]. Since the sucrose-preference test was the only measurement of depressive-like behaviour, additional tests, such as the forced swim test and the tail suspension test could be conducted to validate these results, as done by other aforementioned studies [50, 51, 56, 77]. Accordingly, previous studies have shown that consumption of diets rich in omega-3, vitamin A or vitamin E are able to induce an increase in the levels of hippocampal neurogenesis and hippocampal volume, and reduce depressive symptoms, respectively in adult rodents [94, 95] and humans [96–98]. However, at present, studies investigating the effect of these interventions, especially non-pharmacological, on neurogenesis during adolescence are relatively limited. Further investigations will be of fundamental importance to understand the exact neurogenic mechanisms through which these treatments work in adolescent rodents and, as a consequence, how they can be best used as therapeutic strategies in adolescent humans where putatively similar mechanisms are compromised.

Furthermore, hippocampal neurogenesis, both in adolescence and adulthood, has been mainly investigated at a cellular level thus far, using either histological analyses of hippocampi isolated from rodent tissue [13, 22, 87, 99], or, more rarely, from post-mortem human brain tissue [13, 22, 87, 99]. However, recent advances in the field have made it possible to use neuroimaging tools to measure this process in living humans. Neuroimaging methods, such as Blood Oxygenation Level Dependent-functional MRI, Cerebral Blood Volume and Magnetic Resonance Spectroscopy can be used to relate the putative adult neurogenesis-mediated changes to behaviour, including for aspects of memory and emotion, which are known to be altered by adult neurogenesis in rodent models of depression [100, 101]. However, a major limitation of in vivo neuroimaging investigations is the difficulty in ascribing observed imaging effects to cellular and molecular changes. As such, rodent studies that are of parallel design to the clinical ones are still required in order to assess direct measures of adult neurogenesis which can be linked with neuroimaging outcomes [100, 101]. While at present valid imaging studies assessing hippocampal neurogenesis in adolescents are absent, and very limited in adult humans [100, 101], pre-clinical evidence investigating neurogenesis in adolescent rodents is promising, as demonstrated in this review, and could provide significant cellular and molecular insights, as well as guidance for future neuroimaging investigations in this specific sub-group of individuals.

Although this review has limitations due to the relatively small number of studies, there were a variety of models used, and numerous molecular as well as cognitive and behavioural tests were performed in the studies. This is the first attempt at conducting a *systematic* review summarising changes in hippocampal neurogenesis, hippocampal neuroplasticity, and hippocampal-dependent cognitive function and behavioural outcomes in adolescent rodents exposed to stress models of depression, and also investigating long-term changes in the same outcomes during adulthood. Such a comprehensive insight into the possible holistic effects of neurogenesis is necessary to uncover and translate its potential as a therapeutic target for patients experiencing adolescent depression. While more rodent research is needed to determine whether there is a causal relationship between reduced neurogenesis (induced by a stress challenge) and onset of depressive-like behaviours, it is important to note that out of the 15 studies investigating both neurogenic changes and depressive-like behaviour, 10 studies reported both a decline in neurogenesis or neuroplasticity and concomitant depressive behaviours [48, 51, 60–62, 76, 77, 80, 84, 85]. Of note, while depressive behaviours in rodents are not fully comparable with human depressive symptoms, they still reliably recapitulate some of the aspects of the depressive phenotype often observed in depressed individuals.

Finally, while testing the causal interaction between neurogenesis and behaviour, additional focus should be given to the molecular mechanisms underlying such neurogenic and behavioural modifications, especially when considering the type and duration of the stress paradigms. Also, further examination of sex differences is required. Among the 37 studies included in this review, only 10 look at either female or both male and female rodent models, with only 4 showing differences in findings when comparing male vs female animals [69, 75, 76, 84]. Overall, these findings require validation in order to draw any significant conclusion. In addition, some of the studies included in this review showed high risk of bias as they did not extensively describe the experimental methodologies which were followed. For example, few studies indicate if they blinded or randomised the outcome assessment, therefore suggesting the need for more methodological details in future investigations.

## Conclusion and future directions

In conclusion, this is the first *systematic* review reporting detrimental changes in hippocampal neuronal survival, hippocampal neuroplasticity, and in hippocampal-dependent cognitive function and behavioural outcomes in adolescent rodent models of depression. Much of what is known about the functional role of hippocampal neurogenesis has been studied in adult animals. Given the limited number of studies performed in adolescent animals, more work is needed to elucidate the behavioural effects of changes in hippocampal neurogenesis in adolescence, both in terms of immediate and long-term effects. Moreover, the effect of antidepressants and dietary interventions in adolescence remains to be fully understood. There is the need for novel neuroimaging tools to measure hippocampal neurogenesis in living humans, ultimately bridging the translational gap between animal and clinical findings and contributing to the development of novel and effective treatment approaches targeting hippocampal neurogenesis for adolescents with depression.

### Supplementary information

Supplementary Materials

Supplementary Figure 1

Supplementary Table 1
