# Supplementary material for: Acute and long-term effects of adolescence stress exposure on rodent adult hippocampal neurogenesis, cognition, and behaviour
Source: Mol Psychiatry. 2023 Aug 23;28(10):4124–37. doi: 10.1038/s41380-023-02229-2 (PMC10827658; doi:10.1038/s41380-023-02229-2)
Supplement: Supplementary file 1 — Supplementary Materials [file 41380_2023_2229_MOESM1_ESM.docx]

*Supplementary Materials*

**Acute and long-term effects of adolescence stress exposure on rodent adult hippocampal neurogenesis, cognition, and behaviour**

Alessandra Borsini, PhD^1*#^, Juliette Giacobbe, MSc^1#^, Gargi Mandal, BSc^1^, Maura Boldrini, MD, PhD^2^

^1^ Stress, Psychiatry and Immunology Laboratory, Institute of Psychiatry, Psychology and Neuroscience, Department of Psychological Medicine, King’s College London, UK

^2^ Department of Psychiatry, Columbia University, Division of Molecular Imaging and Neuropathology, New York State Psychiatric Institute, New York, USA.

^#^ These authors contributed equally to this work

* Corresponding Author

Alessandra Borsini, PhD

Stress, Psychiatry and Immunology Lab & Perinatal Psychiatry

Institute of Psychiatry, Psychology and Neuroscience, King’s College London

G.32.01, The Maurice Wohl Clinical Neuroscience Institute

Cutcombe Road, London, SE5 9RT

Tel: 020 7848 0726

Email: alessandra.borsini@kcl.ac.uk

**Supplementary Methods:**

*Inclusion and exclusion criteria*

Included studies had to meet the following criteria: *in vivo* studies in either rats or mice, using a biological or behavioural model of stress-related depression within the adolescence timeframe between PND21-PND65, assessing direct or indirect hippocampal neurogenesis *and* hippocampal-dependent cognitive or depressive-like behavioural outcome in the same period. Keywords for direct measures of neurogenesis included *proliferation*, *differentiation*, or *cell survival*, while indirect measures were searched with *long-term potentiation*, *long-term depression*, *hippocampal volume*, or *synaptogenesis*. In line with the literature, selected hippocampal-dependent cognitive functions included memory, recognition, or pattern separation abilities, while depressive-like behaviour was assessed measuring behavioural despair or anhedonia.

Studies were excluded if they met the following criteria: not in the English language, clinical studies, or in vivo studies not using rats or mice, studies modelling other psychiatric disorders such as schizophrenia, autism, or substance abuse, studies modelling neurological or neurodegenerative conditions, including epilepsy, ischemic stroke, neuropathic pain, Alzheimer’s disease, Parkinson’s disease.

*Search algorithm used*

(((((neurogenesis[Title/Abstract]) OR (progenitor[Title/Abstract]) OR (neuron*[Title/Abstract]) OR (cell survival[Title/Abstract]) OR (cell proliferation[Title/Abstract]) OR (volume[Title/Abstract]) OR (synap*[Title/Abstract]) OR (long-term potentiation[Title/Abstract]) OR (long-term depression[Title/Abstract])) AND ((hippocamp*[Title/Abstract]) OR (dentate gyrus[Title/Abstract]) OR (DG[Title/Abstract]))) OR (((learning[Title/Abstract]) OR (recognition[Title/Abstract]) OR (avoidance[Title/Abstract]) OR (freezing[Title/Abstract]) OR (memory[Title/Abstract]) OR (attention[Title/Abstract]) OR (pattern separation[Title/Abstract]) OR (depression-like[Title/Abstract]) OR (depressive-like[Title/Abstract]) OR (anhedon*[Title/Abstract]) OR (despair[Title/Abstract]) OR (forced swim test [Title/Abstract]) OR (sucrose preference[Title/Abstract]) OR ((model[Title/Abstract]) AND (depress*[Title/Abstract]))) AND ((hippocampus-dependent[Title/Abstract]) OR (hippocamp*[Title/Abstract])))) AND ((((interferon alpha) OR (IFN) OR (cytokin*) OR (inflamm*) OR (LPS) OR (cortisol)) AND (depress*)) OR ((model[Title/Abstract]) AND (depress*[Title/Abstract])) OR ((((chronic) OR (acute) OR (restraint) OR (social)) AND (stress)) OR (isolation)))) AND (((rat) OR (mice) OR (mouse) OR (in vivo) OR (rodent)) AND ((juvenile) OR (adolescen*) OR (puberty) OR (age-dependent)))

*Search process*

Studies published so far until July 2023 were extracted from the electronic databases (PubMed, Embase, PsycInfo, Web of Science) by two of the authors independently (J.G. and G.M.). The results were then compared to ensure reproducibility and accuracy of the algorithm. Subsequently, the extracted studies were filtered for screening and selection by each of the authors, and discussion was carried out in case of disagreement.

*Figure credits*

Figures were created with BioRender.com

**Figure Caption:**

**Supplementary Figure 1.** PRISMA flowchart of search results at each step of the systematic review.

**Supplementary Table 1.** SYRCLE risk of bias assessment of selected studies.
