# Supplementary material for: Acute and long-term effects of adolescence stress exposure on rodent adult hippocampal neurogenesis, cognition, and behaviour
Source: Mol Psychiatry. 2023 Aug 23;28(10):4124–37. doi: 10.1038/s41380-023-02229-2 (PMC10827658; doi:10.1038/s41380-023-02229-2)
Supplement: Supplementary file 2 — Supplementary Figure 1 [file 41380_2023_2229_MOESM2_ESM.docx]

Full-text articles excluded, with reasons
(n = 333)

-Clinical studies, *in vitro,* or *in vivo* studies not using rats or mice

-Studies modelling other psychiatric, neurological, and neurodegenerative conditions.

-Outcomes were measured in late adulthood

-Pre-natal stress model

-Only behavioural outcome was assessed

Records excluded
(n = 535)

Records screened
(n = 905)

Records after duplicates removed
(n = 905)

Identification

Records identified through database searching

(n = 1483)

Eligibility

Screening

Full-text articles assessed for eligibility
(n = 370)

Studies included in qualitative synthesis
(n = 37)

Included
