# Supplementary material for: Acute and long-term effects of adolescence stress exposure on rodent adult hippocampal neurogenesis, cognition, and behaviour
Source: Mol Psychiatry. 2023 Aug 23;28(10):4124–37. doi: 10.1038/s41380-023-02229-2 (PMC10827658; doi:10.1038/s41380-023-02229-2)
Supplement: Supplementary file 3 — Supplementary Table 1 [file 41380_2023_2229_MOESM3_ESM.docx]

| **Study** | **Sequence generation** | **Baseline characteristics** | **Allocation concealment** | **Random housing** | **Blinding**  **(Performance)** | **Random outcome assessment** | **Blinding**  **(Detection)** | **Incomplete outcome data** | **Selective outcome reporting** |
| --- | --- | --- | --- | --- | --- | --- | --- | --- | --- |
| Ago et al., 2014 | + | - | ? | + | - | - | - | - | - |
| Buwalda et al., 2013 | - | - | + | + | + | + | + | ? | ? |
| Coppens et al., 2011 | + | + | + | + | + | + | + | + | - |
| Coutellier et al., 2015 | + | - | NA | + | - | + | - | - | - |
| Dayi et al., 2015 | + | - | + | + | + | + | + | + | - |
| Eiland et al., 2012 | - | - | + | + | + | + | - | - | - |
| Gorbunova et al., 2017 | - | - | - | + | - | - | - | ? | + |
| Gröger et al., 2016 | + | - | + | + | - | + | - | + | + |
| Han et al., 2019 | - | + | + | + | + | - | - | - | - |
| Huang et al., 2012 | + | - | + | + | - | + | + | - | - |
| Huang et al., 2021 | - | + | + | + | + | + | + | + | + |
| Ibi et al., 2008 | - | + | + | + | - | - | - | ? | + |
| Iñiguez et al., 2016 | + | + | + | + | + | + | - | - | - |
| Isgor et al., 2004 | - | + | + | + | + | - | + | + | ? |
| Kovalenko et al., 2014 | + | + | + | + | + | - | + | - | + |
| Lanshakov et al., 2021 | + | - | + | + | + | - | + | ? | - |
| Leussis et al., 2008 | - | + | + | + | + | - | - | - | - |
| Leussis and Andersen, 2008 | - | + | + | + | + | + | - | - | - |
| Li et al., 2017 | - | + | + | + | + | + | + | - | - |
| Li et al., 2019 | - | + | + | + | + | + | + | - | - |
| H.-B. Li et al., 2008 | + | - | + | + | + | + | + | - | - |
| Liu et al., 2015 | + | - | + | + | + | + | + | - | - |
| Maggio et al., 2011 | + | - | + | + | + | + | + | + | - |
| McCormick et al., 2012 | - | + | + | - | - | - | - | - | - |
| McCormick et al., 2010 | - | + | + | + | - | + | - | ? | + |
| Mouri et al., 2018 | + | + | + | + | + | + | + | - | + |
| Nickle et al., 2020 | - | + | + | + | + | + | + | - | ? |
| Oztan et al., 2011 | + | + | + | + | + | - | + | - | + |
| Pawley et al., 2020 | + | + | + | + | + | - | + | ? | - |
| Pinzón-Parra et al., 2019 | + | + | ? | ? | + | - | - | - | - |
| Pisu et al., 2016 | + | - | + | + | + | + | + | + | + |
| Provensi et al., 2019 | - | - | + | + | + | + | + | + | - |
| Sun et al., 2020 | + | + | + | + | + | + | + | - | + |
| Tsoory et al., 2008 | - | - | + | + | + | - | + | + | - |
| Tsoory et al., 2010 | - | - | + | + | + | - | + | + | - |
| Tzanoulinou et al., 2020 | - | - | + | + | + | + | + | - | - |
| Uysal et al., 2012 | + | + | + | + | + | - | + | + | ? |

(+) high risk, (-) low risk, (?) unknown, (NA) Not Applicable
